# Assessment of fragmented QRS formation and its relationship with left ventricular hypertrophy in nonhypertensive acromegaly patients

**DOI:** 10.3906/sag-2101-229

**Published:** 2021-10-21

**Authors:** Muhammet DURAL, Göknur YORULMAZ, Elif Sevil ALAGÜNEY, Kadir Uğur MERT, Ezgi CAMLI, Ahmet Toygar KALKAN, Aysen AKALIN, Nur KEBAPÇI, Ahmet Serdar YILMAZ, Selda MURAT, Belgin EFE

**Affiliations:** 1 Department of Cardiology, Faculty of Medicine, Eskişehir Osmangazi University, Eskişehir Turkey; 2 Department of Endocrinology, Faculty of Medicine, Eskişehir Osmangazi University, Eskişehir Turkey; 3 Department of Endocrinology, Konya Training and Research Hospital, Konya Turkey

**Keywords:** Acromegaly, fragmented QRS, left ventricular hypertrophy, left ventricular mass

## Abstract

**Background/aim:**

It is known that the presence of fragmented QRS (fQRS) on electrocardiography (ECG) is associated with cardiovascular events. The aim of this study was the evaluation of fQRS formation and its relationship with the left ventricular hypertrophy (LVH) parameters in acromegaly patients.

**Materials and methods:**

In total, 47 previously diagnosed with non-hypertensive acromegaly patients and 48 control subjects were included in the study. ECG and transthoracic echocardiography (TTE) were performed for each participant. Acromegaly patients were divided into two groups according to the fQRS formation on the ECG. Left ventricular wall thicknesses, and left atrial diameter (LAD), left ventricular mass (LVM), left ventricular mass index (LVMi), and relative wall thickness (RWT) were obtained.

**Results:**

In control group 5 (10.4%) and in acromegaly group 17 (36.2%) patients had fQRS on ECG (p = 0.003). LAD [36.0 (34.0–38.0) vs. 38.0 (35.0–41.0) mm, p < 0.001], LVM [155.27 ± 27.00 vs. 173.0 (153.0–235.0) g, p < 0.001], LVMi [83.12 ± 13.19 vs. 92.0 (83.0–118.0) g/m², p < 0.001] and RWT [0.39 ± 0.03 vs. 0.43 (0.41–0.45), p = 0.001] were significantly higher in patients with acromegaly. Disease duration was significantly higher (11.59 ± 1.3 vs. 8.2 ± 1.8 years, p < 0.001) in the fQRS (+) group. LAD [41.0 (39.0–42.5) vs. 37.0 (34.7–38.0) mm, p < 0.001], LVM [219.0 (160.5–254.5) vs. 164.0 (153.0–188.0) g, p = 0.017], LVMi [117.0 (92.5–128.5) vs. 86.0 (82.0–100.2) g/m², p = 0.013] and RWT [0.44 (0.42–0.49) vs. 0.43 (0.40–0.44), p = 0.037] were significantly higher in fQSR (+) acromegaly patients. In multivariate logistic regression analysis, disease duration (odds ratio: 10.05, 95% CI: 1.099–92.012, p = 0.041) and LAD (odds ratio: 2.19, 95% CI: 1.030–4.660, p = 0.042) were found to be the independent predictors of fQRS formation.

**Conclusion:**

The results of our study revealed that fQRS (+) acromegaly patients had increased LVH parameters compared to fQRS (-) patients.

## 1. Introduction

Acromegaly is a rare, slowly progressive disease and cardiovascular complications are the most common cause of mortality in these patients [1–3]. The aetiological cause in the majority of patients is growth hormone (GH) secreting pituitary adenoma [4]. As the GH level increases, the insulin-like growth factor-1 (IGF-1) level also increases, leading to IGF-1 receptor activation in cardiac myocytes [5]. mRNA expression increases in sarcomeric proteins, and a subsequent increase in cardiac contractility is seen before hypertrophy and myocardial fibrosis develop [6–9]. Studies have shown that left ventricular mass (LVM) and cardiac wall thickness increase in patients with acromegaly [10–12]. Approximately 16% of patients have cardiac hypertrophy at the time of diagnosis [13]. The standard method used in the evaluation of left ventricular hypertrophy (LVH) is transthoracic echocardiography (TTE). Generally, left ventricular internal dimensions and wall thicknesses are considered when evaluating LVH with TTE. It is also stated that it is more appropriate to use left ventricular mass index (LVMi) to standardize the effect of body size when evaluating LVH in patients with acromegaly [14,15]. LVH is a component of acromegalic cardiomyopathy, which is associated with disease duration, indicative of increased chronic GH/IGF-1 exposure [9]. Detailed evaluation of LVH using different parameters with TTE may be more useful in determining the cardiovascular prognosis. 

Fragmented QRS (fQRS), which is associated with myocardial scar tissue, defined as the presence of an additional R wave (R’), R wave or the S wave notching, or the presence of more than one R’ wave in two consecutive leads in electrocardiography (ECG) [16]. All-cause mortality and cardiac events were found to be associated with the presence of fQRS in coronary artery disease (CAD) patients [17–20]. There are limited data about the relationship between the presence of fQRS in ECG and acromegalic cardiomyopathy. In a recent study, fQRS was found to be associated with impaired left ventricular diastolic functions in acromegaly patients [21]. However, there are no studies that have assessed the association between fQRS and LVH parameters such as LVM, LVMi, and relative wall thickness in patients with acromegaly. As LVH progresses, interstitial fibrosis develops and a deterioration in diastolic and even systolic functions can gradually be seen. Therefore, fQRS, which has been shown to be related to scar tissue, may be associated with LVH in these patients. It suggests that acromegaly patients with LVH may have a higher risk of developing poor cardiovascular outcomes if fQRS is also present. In this study, we aimed to evaluate the presence of fQRS and its relationship with LVH parameters in acromegaly patients. Revealing this relationship may be directive in the follow-up and treatment of acromegaly patients.

## 2. Material and methods 

### 2.1. Study population

A total of 97 patients previously diagnosed with acromegaly in accordance with the American Association of Clinical Endocrinologists (AACE) revised criteria of acromegaly diagnosis who applied to the outpatient clinic of Endocrinology Department between November 2018 and December 2019 were included in the study [22]. ECG and TTE were performed for each patient. Those with hypertension (HT), CAD, severe valvular heart disease, chronic infectious disease, autoimmune disease, renal and/or hepatic failure and patients taking any medication like statins, beta blockers and/or renin-angiotensin-aldosterone system blockers were excluded from the study. Therefore, the study population included a total of 47 acromegaly patients and 48 sex-, body mass index (BMI)-, and age-matched subjects. Firstly, the patient group was compared with the control subjects. Then, the patient population was divided into two groups according to the presence or absence of fQRS on ECG. A detailed cardiovascular and systemic examination was performed in all subjects, and demographic data and anthropometric measures including weight, height, and BMI were recorded. Biochemical measurements fasting blood glucose, total cholesterol, low-density lipoprotein (LDL) cholesterol, serum creatinine, haemoglobin, serum growth hormone (GH), insulin like growth factor 1 (IGF-1) were obtained from all patients with acromegaly. Disease activity was assessed as indicated in the guidelines and those with active acromegaly were identified [23]. After an oral glucose tolerance test (OGTT) (75 g of glucose orally followed by GH measurements every 30 min for 120 min), serum GH measurements were performed previously in every patient at the time of the first diagnosis. The Local Ethics Committee of our institution approved the study protocol.

### 2.1. Electrocardiography 

**Figure 1:**
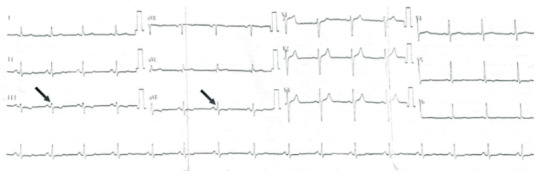
Demonstration of a fQRS (+) acromegalic patient’s ECG. (Black arrows indicate fragmentation).

Two blinded independent cardiologists evaluated the twelve-lead ECGs (filter range, 0.15–100 Hz; AC filter, 50 Hz, 25 mm/s, 10 mm/mV). If there was any discrepancy between the measurements of the two readers, ECGs were examined by a third independent reviewer. All ECGs were scanned and a more detailed analysis was done on a personal computer with × 400% magnification. Fragmented QRS is characterised by the presence of an additional R wave (R’), R wave or the S wave notching, or the presence of more than one R’ wave in two consecutive leads (Figure 1) [16]. 

### 2.2. Echocardiography

As in ECG examination, TTEs were performed by two blinded independent cardiologists by using Vivid S5 with the GE 3S-RS Probe (GE Healthcare). Measurements of the left ventricular internal dimensions, wall thicknesses, and left atrial diameter (LAD) were obtained using linear measurements in para-sternal long-axis view images according to the recommendations of the American Society of Echocardiography [24]. Relative wall thickness (RWT) was calculated as 2 times the left ventricular posterior wall thickness (PWT) divided by the left ventricular end-diastolic diameter (LVEDD) [25]. Left ventricular ejection fraction (LVEF) was measured by using the modified Simpson method (biplane method of disks). LVM was calculated using the formula of the American Society of Echocardiography (ASA) modiﬁed by Devereux et al. [26]. According to the body surface area (BSA), LVMi was calculated. 

### 2.3. Statistical analysis

The distribution of each continuous variable was tested for normality using the Shapiro–Wilk test and is expressed as mean +/- standard deviation (SD). Normally distributed variables were analyzed using the student t-test. Variables with a skewed distribution are expressed as median value [interquartile range (IQR)]. Nonnormally distributed variables were analyzed using the Mann–Whitney U test. The categorical variables are expressed in frequencies and percentages. The Pearson’s Chi-square test was used to compare categorical variables. Multiple binary logistic regression analysis was used to define the independent predictors of fQRS. P values < 0.05 were considered statistically significant. All analyses were performed using the SPSS v: 24.0 software (SPSS Inc., Chicago, IL, USA).

## 3. Results

There was no significant difference in basal characteristics such as age (53.17 ± 13.26 vs. 51.21 ± 12.49 years, p = 0.462), sex (56.3% vs. 51.1% female, p = 0.612), BMI (27.06 ± 3.1 vs. 28.55 ± 4.0 kg/m², p = 0.052) or the number of patients with diabetes mellitus (DM) [7 (14.6%) vs. 8 (17.0%), p = 0.745] between the control group and acromegaly patients. Mean disease duration was 9.43 ± 2.3 years in acromegaly patients. Also, LVEF [65.0 (63.0–67.0) vs. 65.0 (60.0–68.0) %, p = 0.460] and LVEDD [47.39 ± 2.74 vs. 47.0 (46.0–49.0) mm, p = 0.126] were similar between the groups. On the other hand, LAD [36.0 (34.0–38.0) vs. 38.0 (35.0–41.0) mm, p < 0.001], interventricular septum thickness (IVST) [10.0 (9.0–10.0) vs. 10.0 (10.0–12.0) mm, p < 0.001], PWT [9.0 (9.0–10.0) vs. 10.0 (10.0–11.0) mm, p < 0.001], LVM [155.27 ± 27.00 vs. 173.0 (153.0–235.0) g, p < 0.001], LVMi [83.12 ± 13.19 vs. 92.0 (83.0–118.0) g/m², p < 0.001] and RWT [0.39 ± 0.03 vs. 0.43 (0.41–0.45), p = 0.001] were significantly higher in acromegaly group. In control group 5 (10.4%) and in acromegaly group 17 (36.2%) patients had fQRS on ECG (p = 0.003). Baseline characteristics of the control group and the acromegaly patients were shown in Table 1. 

**Table 1:** Baseline characteristics of the study groups.

	Control group(n = 48)	Acromegaly(n = 47)	p
Parameters			
Age, years	53.17 ± 13.26	51.21 ± 12.49	0.462
Body mass index, kg/m²	27.06 ± 3.1	28.55 ± 4.0	0.052
Sex, n (%)			
Female	27 (56.3%)	24 (51.1%)	
Male	21 (43.7%)	23(48.9%)	0.612
Diabetes mellitus	7 (14.6%)	8 (17.0%)	0.745
Disease duration, years	-	9.43 ± 2.3	-
Serum IGF (ng/mL)	-	211.0 (160.0–444.0)	-
Serum GH (ng/mL)	-	2.18 (0.96–5.90)	-
Serum creatinin (mg/dL)	0.80 ± 0.16	0.78 ± 0.15	0.536
Hemoglobin (g/d)	13.83 ± 1.31	13.57 ± 1.55	0.379
LDL-cholestrol (mg/dL)	122.34 ± 32.87	131.58 ± 28.69	0.158
Left ventricular ejection fraction, %	65.0 (63.0–67.0)	65.0 (60.0–68.0)	0.460
Left ventricular end-diastolic diameter, mm	47.39 ± 2.74	47.0 (46.0–49.0)	0.126
Left atrial diameter, mm	36.0 (34.0–38.0)	38.0 (35.0–41.0)	<0.001
Interventricular septum thickness, mm	10.0 (9.0–10.0)	10.0 (10.0–12.0)	<0.001
Left ventricular posterior wall thickness, mm	9.0 (9.0–10.0)	10.0 (10.0–11.0)	<0.001
Left ventricular mass, g	155.27 ± 27.00	173.0 (153.0–235.0)	<0.001
Left ventricular mass index, g/m²	83.12 ± 13.19	92.0 (83.0–118.0)	<0.001
Relative wall thickness	0.39 ± 0.03	0.43 (0.41–0.45)	0.001
Fragmented QRS	5 (10.4%)	17 (36.2%)	0.003

**Figure 2:**
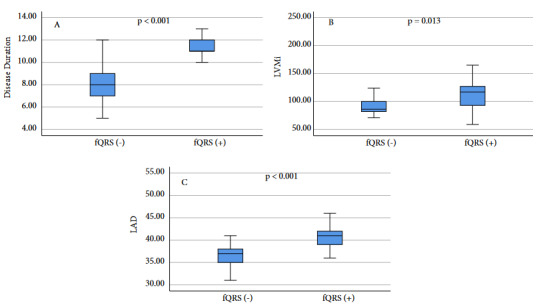
Disease duration, LVMi and LAD were significantly higher in fQRS (+) acromegalic patients compared to fQRS (-). ( LAD left atrial diameter, LMVi left ventricular mass index).

Age (54.00 ± 12.77 vs. 49.63 ± 12.27 years, p = 0.254), sex (47.1% vs. 53.3 female, p = 0.679), BMI (29.2 ± 2.52 vs. 28.17 ± 4.62 kg/m², p = 0.177), the number of patients with DM [4 (23.5%) vs. 4 (13.3%), p = 0.371] and active acromegaly patients [5 (29.4%) vs. 9 (30.0%), p = 0.966] were similar between fQRS (+) and fQRS (-) groups. Disease duration was significantly higher (11.59 ± 1.3 vs. 8.2 ± 1.8 years, p < 0.001) in the fQRS (+) group (Figure 2A). Median serum IGF-1 [208.0 (166.5–372.0) vs. 219.5 (149.2–536) ng/mL, p = 0.599] and GH levels [2.18 (0.78–12.06) vs. 2.13 (1.16–5.24) ng/mL, p = 0.185] were similar between the groups. Also, there is no significant difference in LVEF [65.0 (62.0–68.0) vs. 67.0 (60.0–68.0) %, p = 0.595] and LVEDD [48.0 (45.5–51.0) vs. 47.0 (45.7–48.2) mm, p = 0.440] between fQRS (+) and fQRS (-) patients, while LAD [41.0 (39.0–42.5) vs. 37.0 (34.7–38.0) mm, p < 0.001], IVST [12.0 (10.5–13.0) vs. 10.0 (10.0–11.0) mm, p < 0.001], PWT [11.0 (10.0–12.0) vs. 10.0 (10.0–10.1) mm, p < 0.001], LVM [219.0 (160.5–254.5) vs. 164.0 (153.0-188.0) g, p = 0.017], LVMi [117.0 (92.5–128.5) vs. 86.0 (82.0–100.2) g/m², p = 0.013] and RWT [0.44 (0.42–0.49) vs. 0.43 (0.40–0.44), p = 0.037] were significantly higher in fQSR (+) acromegaly patients (Figures 2B and 2C). A comparison of the clinical, laboratory and echocardiographic parameters of the fQRS (+) and fQRS (-) acromegaly patients is illustrated in Table 2. 

**Table 2:** Clinical, laboratory and echocardiographic parameters of the acromegaly patients.

	fQRS (+)(n:17, 36.2%)	fQRS (-)(n:30, 63.8%)	P
Parameters			
Age (years)	54.00 ± 12.77	49.63±12.27	0.254
Sex, n (%)			
Female	8 (47.1%)	16 (53.3%)	
Male	9 (52.9%)	14 (46.7%)	0.679
Body mass index , kg/m²	29.2 ± 2.52	28.17 ± 4.62	0.177
Diabetes mellitus	4 (23.5%)	4 (13.3%)	0.371
Disease duration, years	11.59 ± 1.3	8.2 ± 1.8	<0.001
Serum IGF (ng/mL)	208.0 (166.5–372.0)	219.5 (149.2–536)	0.599
Serum GH (ng/mL)	2.18 (0.78–12.06)	2.13 (1.16–5.24)	0.185
Active acromgaly n (%)	5 (29.4%)	9 (30.0%)	0.966
Serum creatinin (mg/dL)	0.81 ± 0.16	0.76 ± 0.15	0.311
Hemoglobin (g/dL)	13.39 ± 1.55	13.67 ± 1.56	0.555
LDL-cholestrol (mg/dL)	134.47 ± 34.73	129.89 ± 25.02	0.607
Left ventricular ejection fraction, %	65.0 (62.0–68.0)	67.0 (60.0–68.0)	0.595
Left ventricular end-diastolic diameter,mm	48.0 (45.5–51.0)	47.0 (45.7–48.2)	0.440
Left atrial diameter, mm	41.0 (39.0–42.5)	37.0 (34.7–38.0)	<0.001
Interventricular septum thickness, mm	12.0 (10.5–13.0)	10.0 (10.0–11.0)	<0.001
Left ventricular posterior wall thickness, mm	11.0 (10.0–12.0)	10.0 (10.0–10.1)	<0.001
Left ventricular mass, g	219.0 (160.5–254.5)	164.0 (153.0–188.0)	0.017
Left ventricular mass index, g/m²	117.0 (92.5–128.5)	86.0 (82.0–100.2)	0.013
Relative wall thickness	0.44 (0.42–0.49)	0.43 (0.40–0.44)	0.037

The predictors of fQRS formation were assessed by univariate and multivariate logistic regression analyses. In univariate analysis, disease duration (odds ratio: 4.60, 95% CI: 1.802–11.745, p = 0.001), LAD (odds ratio: 1.62, 95% CI: 1.188–2.217, p = 0.002), IVST (odds ratio: 2.56, 95% CI: 1.397–4.714, p = 0.002), PWT (odds ratio: 4.74, 95% CI: 1.766–12.706, p = 0.002), LVM (odds ratio: 1.01, 95% CI: 1.001–1.025, p = 0.040) and LVMi (odds ratio: 1.03, 95% CI: 1.003–1.058, p = 0.031) were significantly associated with fQRS. Disease duration, LAD, IVST, PWT, and LVMi were included in multivariate logistic regression analysis. Disease duration (odds ratio: 10.05, 95% CI: 1.099–92.012, p = 0.041) and LAD (odds ratio: 2.19, 95% CI: 1.030–4.660, p = 0.042) were found to be the independent predictors of fQRS formation. 

## 4. Discussion

In this study, the relationship between LVH, which is one of the most basic findings of acromegalic cardiomyopathy and fQRS, one of the important findings of myocardial scar tissue, was investigated. The main findings of our study were (i) fQRS was more common in patients with acromegaly than in the control group; (ii) LVH parameters were higher in acromegaly patients than in the control group; (iii) disease duration and LVH parameters were higher in fQRS (+) acromegalic patients than in fQRS (-) individuals; and (iv) disease duration and LAD were the independent predictors of the fQRS formation.

With cardiac magnetic resonance imaging, fQRS has been shown to be associated with scar tissue in many cardiac diseases [27–30]. In patients with acromegaly, chronic excess of GH and IGF-1 secretion causes biventricular concentric hypertrophy and then interstitial fibrosis develops, which is accompanied by increased extracellular collagen deposition, lympho-mononuclear infiltrations, and myofibrillar derangement [31–33]. Due to these areas of fibrosis, these patients are expected to have a higher rate of fQRS formation on ECG than healthy individuals. Indeed, in our study, fQRS formation was higher in patients with acromegaly than in the control group. 

The most important factor that determines the prognosis in patients with acromegaly is cardiac involvement because approximately 60% of these patients die due to cardiovascular diseases [3]. Initially, biventricular hypertrophy develops, and later diastolic and systolic functions are impaired [34]. It has been shown in many studies that LVM is associated with adverse cardiac outcomes [35,36]. LVH is seen in approximately 16% of patients with acromegaly [13]. When evaluating LVH, not only wall thicknesses, but also LVM and even LVMi should be taken into consideration. Therefore, LVM, LVMi and RWT parameters were evaluated in addition to left ventricular wall thicknesses in our study. We showed that LVM, LVMi, and RWT were significantly higher in acromegaly patients than in the control group, besides left ventricular wall thicknesses. 

In our study, we analysed patients with acromegaly by dividing them into two groups based on the presence of fQRS. We found that disease duration was significantly longer in the fQRS (+) group. Chronic exposure to excess GH and IGF-1 causes interstitial fibrosis in the myocardium [31]. Therefore, more fibrosis will occur in patients with longer disease duration. As a result, fQRS will also be more likely to occur in patients with longer disease duration. Also, the LVH parameters such as IVST, PWT, LVM, LVMi, and RWT were found to be significantly higher in fQRS (+) acromegaly patients than in fQRS (-) individuals. It has been shown in many studies that LVM is a predictor of cardiovascular disease [35,37]. In a previous study, 15 nonhypertensive acromegaly patients were compared with healthy control subjects and it was found that acromegaly patients had increased LVMi, RWT and impaired diastolic functions [38]. Also, with treatment, it was shown that both LVH parameters regressed and diastolic functions improved [38]. The regression in LVH and the improvement in myocardial function together with treatment shows the relationship between them. The fact that LVH parameters were increased in acromegaly patients with fQRS (+) in our study may indicate that these patients have more advanced acromegalic cardiomyopathy. Dereli et al.[21] evaluated fQRS frequency and LV functions in 60 acromegaly patients in their study. As in our study, it was found that the disease duration was higher in the group with fQRS (+) [21]. Those with fQRS (+) have been shown to have a greater impairment in LV function parameters such as *E\A* ratio, *E’* velocity, *E/E’* ratio, isovolumic relaxation time, and myocardial performance index (MPI) [21]. They also found that fQRS is an independent predictor of MPI associated with LV dysfunction [21]. As a result, the researchers stated that fQRS may be an indicator of acromegalic cardiomyopathy [21]. Differently, in our study, the relationship between LVH parameters and fQRS was evaluated and patients with HT were excluded. Our results are supportive and complementary to previous studies. Increased hypertrophy means increased fibrosis and scar tissue, and, in this case, fQRS can be thought to occur more often. In addition, we found that acromegalic patients with fQRS (+) had a larger left atrium than those with fQRS (-). In the multivariate regression analysis, one of the independent predictors of fQRS was the LAD. Atrial dilatation, which is a sign of left atrial remodeling and diastolic dysfunction, has been shown to be associated with many cardiovascular diseases such as atrial fibrillation and heart failure [39,40]. Therefore, those with fQRS may have greater myocardial dysfunction, and these patients should be followed more closely to prevent the development of advanced acromeglic cardiomyopathy. In fQRS (+) acromegalic patients, using speckle tracking echocardiography and cardiac magnetic resonance imaging techniques can provide additional benefits in follow-up. 

Our study is the first study assessing the relationship between the presence of fQRS and its relationship between the LVH parameters in non-hypertensive acromegaly patients. However, our study has several limitations. This is a single center, cross sectional study and the number of patients in the study is relatively small. Since LVH parameters were evaluated, only non-hypertensive patients were included in the study. In addition, more advanced techniques such as speckle tracking echocardiography and cardiac magnetic resonance imaging to evaluate the myocardial functions and extent of scar tissue could be used. Also, the lack of long-term follow-up in order to assess the clinical significance of fQRS formation in acromegaly patients was the other limitation of our study. Large scale, prospective, long-term studies are needed to clarify the clinical outcomes of fQRS formation in acromegalic patients. 

## 5. Conclusion

In conclusion, we showed that fQRS (+) acromegaly patients had increased LVH parameters and LAD than fQRS (-) individuals. Disease duration and LAD were the independent predictors of fQRS formation. Therefore, fQRS (+) acromegaly patients should be followed more closely for acromegalic cardiomyopathy and cardiovascular diseases.

## Informed consent

This study has been approved by Eskisehir Osmangazi University Ethics Committee (Approval no. 25403353-050.99-E.46782) and was performed in accordance with the ethical standards laid down in the 1964 Declaration of Helsinki and its later amendments. All participants provided informed consent in the format required by the ethics committee
